# Special Issue “Phytohormones 2022–2023”

**DOI:** 10.3390/biom14091146

**Published:** 2024-09-11

**Authors:** Guzel Kudoyarova, Guzel Akhiyarova

**Affiliations:** Ufa Institute of Biology, Ufa Federal Research Centre of the Russian Academy of Sciences, Pr. Octyabrya, 69, 450054 Ufa, Russia; akhiyarova@rambler.ru

The hormonal system plays a decisive role in controlling plant growth and development. Alongside classical plant hormones (auxins, cytokinins, gibberellins, and abscisic acid), hormonal function is now attributed to jasmonates (JAs) and salicylic acid. Plant hormones are capable of influencing such vital processes as plant growth and development [1], productivity [2], adaptation to the environment [3], and resistance to biotic [4] and abiotic stresses [5], among others, and they have become attractive biotechnological tools aimed at improving plant performance for human needs. The synthesis of plant hormones by rhizosphere microorganisms is the basis for their capacity to promote plant growth as well as for the application of their preparations in crop production [6]. Still, the successful use of plant hormones depends on understanding the mechanisms of their action. This Special Issue of Biomolecules examines different aspects plant hormones (cytokinins [7,8,9,10], abscisic acid (ABA [7,10,11,12]), auxins [8,13], salicylic acid [14], and jasmonates [14,15,16,17]) play in controlling processes involved in pollen germination [12] and self-incompatibility [9], nitrogen use efficiency [13], the recovery of gas exchange after water stress relief [7], the effects of planting density and rhizosphere bacteria on growth of plants [11], plant resistance to heavy metals [16] and blast disease [17], the regulation of fungal growth, development, and virulence [10], and the accumulation of biomass and secondary metabolites in vitro [8,14,15].

The study conducted by Breigina et al. [12] revealed the presence of ABA in the stigma exudates of *Nicotiana tabacum* and *Lilium longiflorum* and characterized the fatty acid composition in them. Stigma, which is the terminal part of the pistil in flowering plants, receives pollen, ensures its germination, and directs pollen tubes (PT) to the style [18]. The exact composition of the stigma exudates is still unknown because only some components have been studied. High-performance liquid chromatography-mass spectrometry revealed the presence of ABA in the stigma exudates, and its physiological significance was confirmed by *in vitro* experiments showing that the ABA concentration found in the exudate strongly stimulated the germination of tobacco pollen.

The article by Zakharova et al. [9] examined programmed cell death as a self-incompatibility factor, that is, a mechanism that prevents the fertilization of ovules by its own pollen. This hypothesis is based on earlier studies (e.g., [19] and Zakharova et al. [9]), which provided further evidence in support of this hypothesis in petunia and tomato. It was shown that, before a compatible pollination, treatment with exogenous cytokinin activated caspase-like proteases involved in the control of programmed cell death [20] and slowed down the germination and growth of petunia and tomato male gametophytes both in vitro and in vivo. In the case of self-incompatible pollination, the activity of the *IPT5* gene, which controls cytokinin synthesis, was increased, and the expression of the cytokinin oxidase genes controlling cytokinin catabolism was significantly down-regulated. The authors believe these results suggest a critical role played by cytokinins in the mechanism underlying self-incompatibility.

Lebedev et al. [13] aimed to elucidate the interaction between phytohormones and nutrients in woody plants. The authors measured hormone levels in transgenic birch plants expressing the pine glutamine synthetase gene *GS1* during rooting in vitro and budburst under outdoor conditions. The glutamine synthetase gene was chosen due to its important role in nitrogen assimilation in plants [21]. It was shown that auxin and ABA contents measured in the plants during rooting, dormancy, and budburst depended both on nitrogen availability and transgenic status. Changes in the levels of auxin and ABA are discussed with regard to budburst and rooting.

The article by Zlobin et al. [7] addressed the mechanisms underlying the delayed or incomplete recovery of gas exchange after drought relief that limits assimilation in the post-drought period. The accumulation of ABA, which can close stomata, and decrease in the level of cytokinins, which can keep stomata open, are known to control stomatal conductance during water deficiency [22]. It was suggested that restoring the ABA/cytokinin ratio should help normalize water relations after drought relief, while sustained ABA accumulation is thought to be the main cause of delayed post-drought gas exchange recovery [23]. This suggestion was refuted by the present study showing that post-drought stomatal conductance in Scots pine needles remained suppressed for 2 weeks after plant re-watering, while ABA levels decreased, and cytokinin/ABA ratios recovered rapidly in the re-watered plants. It was concluded that the delay in the recovery of stomatal conductance was not due to the sustained accumulation of ABA or decline in the cytokinin/ABA ratio but because of some other causes.

Vysotskaya et al. [11] demonstrated that the growth-inhibitory effect of increased planting density can be reduced by abscisic acid-degrading bacteria. The application of high-density planting can increase crop productivity per unit area of cultivated land. However, the application of this technique is limited by the inhibition of plant growth in the presence of neighbors, which is not only due to their competition for resources but is also caused by growth regulators. As previously shown, ABA accumulated in plants under increased planting density, inhibiting their growth [24]. The present research showed that inoculation of the lettuce rhizosphere with a strain of *Pseudomonas plecoglossicida* 2.4-D capable of degrading ABA positively affected the growth of grouped plants, reducing the negative effects of competition. The ABA concentration increased in the presence of neighbors both in soil and plant shoots, but its accumulation, leading to inhibition of the growth of grouped plants, was prevented by bacteria. The results confirm the role of ABA in the response of plants to the presence of competitors and the possibility of reducing the negative effect of competition on plant productivity with the help of bacteria capable of degrading this hormone.

The article by Repkina et al. [16] focused on the capacity of methyl jasmonate to increase resistance of wheat plants to cadmium stress. Specifically, the authors suggested that the changes in composition of fatty acids under the influence of external chemical agents (MJ and Cd) affected the integral packing of membranes. The stress-induced increase in palmitic acid can be considered a biochemical mechanism of adaptation of the lipid metabolism to heavy metals, important for the maintenance of cellular biomembrane fluidity.

Su et al. [17] reported the results of a study of resistance to blast disease caused by the fungal pathogen *Magnaporthe oryzae* in rice plants bearing the knockout construct of lipoxygenase3 (LOX3), an enzyme involved in JA synthesis [25]. The LOX3 knockout reduced the defense response, while exogenous JA treatment alleviated blast symptoms in the infected plants by hindering hyphal expansion, inhibiting ROS-mediated cell death, and increasing the defense response. At the same time, the knockout of LOX3 improved rice quality. It is suggested that exogenous JA provides a means to compensate for the reduction in the defense responses of LOX3 knockout rice lines, suggesting potential applications in agricultural production.

The role of CKs and ABA in regulation of the growth of pathogenic fungus *Stagonospora nodorum* Berk, its development, and virulence is addressed in the article by Nuzhnaya et al. [10]. The authors compared the ability of two virulent isolates and one avirulent isolate of the fungus to synthesize three groups of hormones (CKs, ABA, and auxins) and studied the effect of exogenous ABA and zeatin on growth, sporulation, and gene expression of necrotrophic effectors (NEs) and transcription factors (TFs) in them. The effect of these hormones on the above characteristics was shown to depend on both the genotype of the isolate and the concentration of hormones and was associated with the regulation of carbohydrate metabolism.

Effects of auxin (indole-3-butyric acid) and jasmonic acid on the formation of adventitious roots of *Acmella radicans* were studied in vitro by Bernabé-Antonio et al. [14]. It was demonstrated that an *A. radicans* root culture is capable of producing secondary metabolites, while their production and antioxidant activity can be enhanced using jasmonic acid.

The topic of the influence of plant hormones on the production of secondary metabolites in vitro was continued in the article by Grzegorczyk-Karolak et al. [8] using *Salvia bulleyana*, a plant native to the Chinese Yunnan Province. Auxin indole-3-acetic acid and several cytokinins were added to a culture medium to establish an effective system for propagating *S. bulleyana* shoots and obtain large amounts of material rich in bioactive compounds. In the study, 0.1 mg/L IAA and 1 mg/L meta-topoline were found to be the most efficient for polyphenol accumulation and can be used for the production of medicinally relevant compounds.

The effects of methyl jasmonate (MeJ) on growth and taxoid formation in the cell culture of *Taxus wallichiana* were reported in the article by Demidova et al. [15]. The characteristics of the «young» and “old” suspension cultures were compared. MeJ addition to the “young” culture caused a 10-fold increase in the C13-OH taxoid production (up to 0.12–0.19 mg/gDW, comparable to the bark of yew trees). When added to the “old’ culture, it notably increased the content of C14-OH taxoids. These findings suggest that hormonal signaling in dedifferentiated yew cells grown in vitro is different from that in planta and can be affected by the culture’s age, which leads to the predominant formation of C14-OH taxoids versus C13-OH taxoids and a modified cell response to exogenous MeJ treatment.
